# Learning to Solve Trigonometry Problems That Involve Algebraic Transformation Skills via Learning by Analogy and Learning by Comparison

**DOI:** 10.3389/fpsyg.2020.558773

**Published:** 2020-09-25

**Authors:** Bing Hiong Ngu, Huy P. Phan

**Affiliations:** School of Education, University of New England, Armidale, NSW, Australia

**Keywords:** analogical learning, learning by comparison, linear equations, trigonometry problems, cognitive load

## Abstract

The subject of mathematics is a national priority for most countries in the world. By all account, mathematics is considered as being “pure theoretical” (Becher, 1987), compared to other subjects that are “soft theoretical” or “hard applied.” As such, the learning of mathematics may pose extreme difficulties for some students. Indeed, as a pure theoretical subject, mathematics is not that enjoyable and for some students, its learning can be somewhat arduous and challenging. One such example is the topical theme of *Trigonometry*, which is relatively complex for comprehension and understanding. This Trigonometry problem that involves algebraic transformation skills is confounded, in particular, by the location of the pronumeral (e.g., *x*)—whether it is a numerator sin30° = *x*/5 or a denominator sin30° = 5/*x*. More specifically, we contend that some students may have difficulties when solving sin30° = *x*/5, say, despite having learned how to solve a similar problem, such as *x*/4 = 3. For more challenging Trigonometry problems, such as sin50° = 12/*x* where the pronumeral is a denominator, students have been taught to “swap” the *x* with sin30° and then from this, solve for *x*. Previous research has attempted to address this issue but was unsuccessful. *Learning by analogy* relies on drawing a parallel between a learned problem and a new problem, whereby both share a similar solution procedure. We juxtapose a linear equation (e.g., *x*/4 = 3) and a Trigonometry problem (e.g., sin30° = *x*/5) to facilitate analogical learning. *Learning by comparison*, in contrast, identifies similarities and differences between two problems, thereby contributing to students’ understanding of the solution procedures for both problems. We juxtapose the two types of Trigonometry problems that differ in the location of the pronumeral (e.g., sin30° = *x*/5 vs. cos50° = 20/*x*) to encourage active comparison. Therefore, drawing on the complementary strength of learning by analogy and learning by comparison theories, we expect to counter the inherent difficulty of learning Trigonometry problems that involve algebraic transformation skills. This conceptual analysis article, overall, makes attempts to elucidate and seek clarity into the two comparative pedagogical approaches for effective learning of Trigonometry.

## Introduction

The topic of Trigonometry is part of secondary mathematics curriculum. Trigonometry is prerequisite knowledge for learning Calculus in senior mathematics, and is essential for students who wish to pursue Science, Technology, Engineering, and Mathematics (STEM) courses. Learning Trigonometry problems requires an understanding of multiple interrelated mathematical concepts, such as *algebraic transformation skills*, *geometry knowledge*, and *reasoning of graphical representation of concepts*. Owing to the need to learn multiple interrelated concepts, students experience great difficulty when learning Trigonometry problems (Blackett and Tall, 1991; Kendal and Stacey, 1998). Our objective in this conceptual analysis article is to highlight the importance of scaffolding algebraic transformation skills to facilitate the initial phase of learning Trigonometry problems. The main focus here is to calculate the unknown side of a right-angled triangle from a known side and an angle, which can pose a challenge for many students. More specifically, we contend that some students may have difficulties when solving sin30° = *x*/5, say, despite having learned how to solve a similar problem, such as *x*/4 = 3. For more challenging Trigonometry problems, such as sin30° = 12/*x* where the pronumeral is a denominator, students have been taught to “swap” the *x* with sin30° and then from this, solve for *x* (Source: personal communication).

We argue that such teaching strategy, as detailed, for Trigonometry problems falls short of addressing the algebraic transformation skills, which are required to solve Trigonometry problems. It does not, for example, attempt to relate a student’s prior knowledge of solving *linear equations with a fraction* to the solving of Trigonometry problems. Apart from this, students may experience great difficulty when they attempt to distinguish the difference between two types of Trigonometry problems that look similar, but yet are conceptually different from each other, consequently because of the relative position of the pronumeral (i.e., a numerator vs. a denominator) (Kendal and Stacey, 1998). On this basis, it is important for educators to consider different theoretical approaches, pedagogical strategies, and/or educational programs that could help students acquire relevant skills to solve Trigonometry problems that differ in terms of the location of the pronumeral (i.e., a numerator vs. a denominator). One possibility, for example, is related to the use of different, but comparable learning theories that could facilitate effective learning and inform meaningful understanding. The aim of this article then, situated within the context of the topical theme of Trigonometry, is for us to examine the effectiveness of two learning theories: *learning by analogy* and *learning by comparison*. This analysis, we contend, could form the basis for further research development, theoretically, empirically, conceptually, and/or methodologically, into the effective application of different learning theories.

## The Concept of Learning by Analogy

*Learning by analogy*, underpinned by *structure mapping theory* (Gentner, 1983), has provided a theoretical framework for research development into the study of word problems (Reed et al., 1985, 2012; Reed, 1987; Ross and Kennedy, 1990; Cummins, 1992). The structure mapping theory emphasizes the construction of “relational commonalities” between a source example (a learned problem) and a target problem (a new problem) in terms of problem structure. Two-word problems may have different problem contexts but share a similar problem structure, for example: (i) “If 20% of my saving is $300, what is my saving?” vs. (ii) “Joshua pays $260 a week for the rent and this represents 25% of his weekly wages. How much does Joshua earn a week?” Using the *Algebra approach*, we can set up two equations, such as 20%*x* = $300 and 25%*x* = $260, respectively, and solve for *x*. Because these two equations share relational elements, they share the same solution procedure. Analogical transfer is likely to occur if learners can successfully map relational elements between a source example and a target problem. Indeed, analogical reasoning of a learned problem and a new problem enables learners to retrieve the schema for the learned problem, which is applicable for solving the new problem.

Holyoak (1984) and Holyoak and Koh (1987) highlighted four tasks to facilitate analogical learning: construct a mental representation of the source example and the target problem (Task 1), retrieve the source example as an analog to the target problem (Task 2), map the relational elements of the source example and the target problem (Task 3), and extend the mapping to solve the target problem (Task 4). The authors neither suggested a definite sequence to implement these four tasks, nor indicated which task or tasks are critical for fostering analogical learning.

Research has reported the benefit of including *supportive cues*, such as a hint (Novick and Holyoak, 1991) or a reminder (Ross, 1984) to access the source example. Thus, the provision of a hint addresses Task 2. In a study conducted by Cummins (1992), practice in extracting similar concepts between the source example and the target problem resulted in analogical transfer. We can attribute the extraction of similar concepts to mapping activities, which addresses Task 3. Other researchers have also emphasized the mapping process to achieve analogical transfer (Gentner et al., 2003). Participants who completed a diagram highlighting relational elements between two negotiation scenarios outperformed those participants who merely studied the two negotiation scenarios. In contrast, however, Reed (1989) failed to find evidence of analogical transfer for word problems despite having addressed Tasks 2 and 3: (1) provided a hint for students to access the source example, (2) required students to construct concept-mapping tasks between the source example and the target problem.

A study by one of us some years back (Ngu and Yeung, 2012) revealed that the presence of multiple components in the source example (i.e., symbolic equations, categorization) or the target problem (i.e., a hint, categorization), or both, actually facilitated the mapping of symbolic equations in the source example onto the target problem, resulting in the effectiveness of analogical transfer. The findings, we contend, have provided new theoretical insights into learning by analogy by emphasizing the importance of having multiple components, rather than a single component to foster analogical transfer for word problems.

In light of prior studies on learning by analogy, the use of hints to access the source example appears to be a critical analogical task to facilitate transfer. Nonetheless, using a hint to access the source will become redundant if the source example is kept visible, while learners engage in mapping the source example and the target problem (Richland et al., 2007). A number of researchers (Gentner et al., 2003; Rittle-Johnson and Star, 2007; Richland and McDonough, 2010) have noted the advantage of presenting two examples simultaneously rather than sequentially. Presenting two examples simultaneously, in this case, eliminates the need to provide a hint for learners to access the source example. Presenting examples in a sequential manner, in contrast, requires a possible need to provide appropriate cues to remind learners of the source example.

Indeed, the juxtaposition of two worked examples not only renders the retrieval of source example unnecessary, but it also provides opportunity for learners to engage in effortful comparison. In their study, Kurtz et al. (2001) advocated the implementation of mutual alignment to foster the abstraction of the underlying common structure across two partially understood scenarios. Participants who jointly interpreted the two scenarios in conjunction with listing specific correspondences demonstrated greater mutual alignment than those participants who either jointly or separately interpreted the two scenarios. Furthermore, engaging in mutual alignment between two partially understood text-based examples promoted analogical transfer of a complex science concept (Orton et al., 2012).

Clearly, from the preceding sections, research has supported the use of the juxtaposition of two examples to promote analogical learning. Nonetheless, the efficiency of performing one-to-one mapping activities depends on the orientation of the two images (Kurtz and Gentner, 2013), or the objects in two examples (Matlen et al., 2020). Aligning two examples in the same orientation instead of different orientations, in this case, facilitates direct alignment of the mapping process, which improves the efficiency of analogical reasoning.

## The Concept of Learning by Comparison

Building on the structure mapping theory (Gentner, 1983) to foster analogical transfer, a number of studies recently highlighted the positive effects of *learning by comparison* (Alfieri et al., 2013; Ziegler and Stern, 2014; Rittle-Johnson et al., 2017). For example, Durkin and Rittle-Johnson (2012) investigated the effect of comparing correct and incorrect examples for learning Decimal Numbers. Displaying correct decimal and incorrect decimal concepts simultaneously helped students rectify their misconceptions regarding the magnitude of decimal numbers. A similar line of research involved asking students to justify why a specific solution step was a good step (e.g., 1 = 2*x* − 5, 6 = 2*x*), or a wrong step (e.g., 3 = 6*x* − 2, 3 = 3*x*), helped students consolidate and refine their understanding of conceptual knowledge, which was involved in solving linear equations (Booth et al., 2013). Moreover, Große and Renkl (2007) demonstrated the positive effect of using correct and incorrect worked example in the domain of Probability Problems. They argued that learning from correct and incorrect examples offers learners with the opportunity to distinguish similarities and differences between the two types of worked examples.

Instead of comparing correct and incorrect worked examples to facilitate mathematics learning, comparing two contrasting Algebra expressions (e.g., *y*^3^ + *y*^3^ = 2*y*^3^ vs. *y*^3^ × *y*^3^ = *y*^6^) side-by-side also helped students to differentiate superficially similar (e.g., letter, number), but conceptually different concepts (e.g., addition vs. multiplication) within two contrasting worked examples (Ziegler and Stern, 2014). Students who studied contrasting Algebra expressions simultaneously outperformed those students who studied algebra expressions sequentially. Overall, then, research development to date has affirmed the benefit of using learning by comparison to enhance effective mathematics learning.

## Learning by Analogy and Learning by Comparison in Mathematics Classroom

Apart from conducting laboratory testing, researchers have also examined cross-national differences when using learning by analogy in mathematics lessons for eight-grade students (Richland et al., 2007). Teachers in high performing countries in mathematics (e.g., Hong Kong, Japan), for example, tend to use far more visual-spatial supports and linking gestures to emphasize analogical comparisons than their U.S. counterparts. Frequent use of visual-spatial supports and linking gestures that direct students’ attention to the source analog may help reduce cognitive processing demands, as it eliminates the need to search for the source analog (Richland et al., 2017).

Instead of conducting learning by comparison in intact classrooms that lasted a few days, Star et al. (2015), in contrast, implemented a 1-year intervention between “comparison” curriculum and “business as usual” curriculum. The comparison curriculum was incorporated into regular curriculum as supplementary materials. Greater use of comparison materials correlated with higher gain in procedural knowledge. Nonetheless, though, it was a challenge to encourage teachers to use comparison materials consistently throughout the year.

Indeed, from the above, analogical reasoning is facilitated by the use of supporting cues (e.g., a hint) to draw learners’ attention to a relevant source example that shares a solution procedure, which is similar to the target problem. Nevertheless, we could exclude a hint to access the source example if we place the source example and the target problem side-by-side (e.g., Rittle-Johnson and Star, 2007). Mapping relational commonalities between the source example and the target problem is another critical analogical task that facilitates analogical transfer. However, successful analogical transfer depends on active comparison process (e.g., joint interpretation plus list specific correspondences) (e.g., Kurtz et al., 2001) and direct alignment of the examples (e.g., Matlen et al., 2020). It is interesting to note though, from our examination of the literature, that constructing a mental representation of the source example in the initial phase of learning by analogy (Holyoak and Koh, 1987) has received minimal attention in terms of research and/or teaching development.

The salient point of learning by comparison, in contrast, is the simultaneous display of two worked examples side-by-side, which then allows learners to identify similarities and differences between the two solution procedures of the two worked examples (Rittle-Johnson et al., 2017). Consequently, learning by comparison has the potential to strengthen a learner’s understanding of mathematical concepts (or misconceptions), as well as specific procedures that are relevant to the two worked examples (e.g., Booth et al., 2013).

It is interesting to note that, methodologically, learning by analogy and learning by comparison have consisted of the use of different types of interventions. In relation to learning by analogy, researchers have implemented interventions in laboratory and classroom settings (Alfieri et al., 2013) and have, likewise, included the use of visual-spatial supports and linking gestures (Richland et al., 2007). In a similar vein, for learning by comparison, researchers have conducted both short-duration interventions (e.g., Rittle-Johnson and Star, 2007) and long time-duration interventions (e.g., one calendar year) to enhance the learning of Algebra (Star et al., 2015). Overall, then, we contend that pedagogical practices that incorporate the use of both learning by analogy and learning by comparison are effective, helping to facilitate students’ learning of mathematics. Which approach is more appropriate and/or effective? From our point of view, we acknowledge that the two pedagogical approaches are complementary with each other—the strength of one approach may counter the weakness of the other approach and as such, this “complementary balance” may reflect a holistic position when one learns how to solve Trigonometry problems.

## Trigonometry Problems

Our objective for discussion is to propose effective instruction that could facilitate the learning of two different types of Trigonometry problems, which differ because of the relative position of the pronumeral—for example, cos60° = *x*/2, where the pronumeral is a numerator, and sin30° = 8/*x*, where the pronumeral is the denominator. As noted earlier, Trigonometry problems are analogous to linear equations that have a fraction. We have found from our research that it is more difficult to solve linear equations that have a fraction, especially where the pronumeral is a denominator instead of a numerator because the former involves more solution steps (Ngu and Phan, 2016).

Despite the importance of Trigonometry problems in secondary school mathematics curriculum, research pertaining to effective teaching and learning of this problem type is relatively scant (Kendal and Stacey, 1998; Weber, 2005; Weber et al., 2008). Research has indicated that students experienced great difficulty when they have to learn how to solve both types of Trigonometry problems (e.g., sin30° = 8/*x* vs. cos60° = *x*/2) (Kendal and Stacey, 1998). To address the issue, Kendal and Stacey (1998) compared the *unit circle* method and the *ratio* method with a particular focus to address students’ difficulty in applying algebraic transformation skills to solve Trigonometry problems with pronumerals as denominators. For the unit circle method, the authors created a right-angled triangle that shared similar properties to a given right-angled triangle. Several skills were required to generate a scale factor, which would enable the solving of Trigonometry problems with pronumerals as denominators (e.g., aligned two right-angled triangles in terms of similar properties). For the ratio method, in contrast, on the basis of information provided in a right-angled triangle, students were required to express the trigonometric ratio in an equation (e.g., cos60° = *x*/2), and then solve for *x*. The post-test results revealed that the unit circle method was inferior to the ratio method, irrespective of the type of Trigonometry problems (i.e., sin30° = 8/*x* or cos60° = *x*/2).

It is well-known that learning how to solve Trigonometry problems that involve algebraic transformation skills is a pervasive issue, which continues to persist for many secondary school students (Weber, 2005). This difficulty, perhaps, is confounded by existing instructional materials that are described and recommended in textbooks (e.g., Vincent et al., 2012). For example, Vincent et al. (2012) detailed the solution procedure for Trigonometry problems that have pronumerals as numerators (e.g., cos50° = *x*/8): multiply both sides by 8, which involves one operation. When the pronumeral is a denominator (e.g., sin30° = 12/*x*), in contrast, the authors recommended two operations: (i) multiply both sides by *x*, and (ii) divide both sides by sin30°. We contend that the presentation of the solution procedure for both types of Trigonometry problems is logical. Having said this, however, we note that Vincent et al. (2012) did not make an attempt to relate the two types of Trigonometry problems to students’ prior knowledge of linear equations with a fraction.

We argue that it is important to consider the extent to which learning by analogy, which may draw on a learner’s prior knowledge of solving linear equations with a fraction, could facilitate the effective solving of Trigonometry problems that involve algebraic transformation skills. At the same time, we also consider the potency of learning by comparison to discern different solution procedures for Trigonometry problems that have pronumerals as denominators (e.g., sin30° = 8/*x*) or as numerators (e.g., cos60° = *x*/2). We will discuss the solution procedure of linear equations with a fraction in the next section, given that they are related to Trigonometry problems.

### Solution Procedure of Linear Equations

**FIGURE 1:**
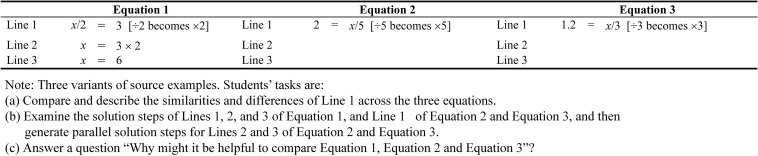
Three variants of source examples.

In line with prior studies (e.g., Ngu et al., 2015; Ngu and Phan, 2016), we use *relational* and *operational* lines to describe the solution procedure of a linear equation. A relational line refers to the “quantitative relation where the left side of the equation is equaled to the right side of the equation.” An operational line, in contrast, refers to the use of an “operation that alters the state of the equation and, hence, such a procedural step would preserve the equality of the equation.” For example, referring to Eq. 1 in Figure 1, Lines 1 and 3 are relational lines whereas, in contrast, Line 2 is an operational line. Moreover, for this example, we use the *inverse method* to illustrate the solution procedure of equations that have a fraction (Figure 1). Our previous research has affirmed the use of the inverse method rather than the balance method for solving linear equations, especially those equations that involve multiple solution steps (Ngu et al., 2015, 2018). The main difference between the inverse method and the balance method, in this sense, lies in the operational line (e.g., × 4 on both sides vs. ÷ 2 becomes × 2) (see Figure 1 for the inverse method). Central to the nature of the inverse method is the inverse operation itself. The conceptualization of the inverse operation of division, in this case, is that of multiplication (i.e., ÷ 2 becomes × 2). According to Ding (2016), interestingly, understanding inverse operation in the primary school years is likely to assist with senior mathematics studies (e.g., differentiation and integration in calculus). The inverse method, as we have found from our existing research, is likely to impose lower cognitive load than the balance method, especially for linear equations that have multiple solution steps.

## Trigonometry Problems With a Pronumeral as the Numerator

This section of the article details our fundamental premise, which “equates” a Trigonometry problem that has a pronumeral as a numerator (e.g., sin30° = *x*/6) with that of a linear equation with a fraction (e.g., *x*/4 = 3). Capitalizing on existing research investigations (Holyoak, 1984; Holyoak and Koh, 1987; Kurtz et al., 2001; Ngu and Yeung, 2012; Alfieri et al., 2013; Rittle-Johnson et al., 2017; Matlen et al., 2020), we propose two major stages to facilitate analogical learning for Trigonometry problems that involve algebraic transformation skills. We now discuss each of the stages below in detail.

### First Stage: Three Variants of Source Examples

According to curriculum development and timetable scheduling, we assume that students would have learned linear equations with a fraction before they learn the topic of Trigonometry (Vincent et al., 2012). This sequencing is advantageous as it enables educators to draw parallel between a learned problem, such as a linear equation with a fraction, *x*/4 = 3 (a source example) and a new problem, such as a Trigonometry problem, sin30° = *x*/6 (a target problem). Novice learners, however, may not necessarily recognize the similarity between the source example and the target problem without a teacher’s scaffolding. To facilitate analogical learning, the first stage involves a mental representation of three variants of source examples in terms of a solution procedure (Figure 1). The aim, in this case, is to assist learners to select a relevant source example from three different variants of source examples, which then could serve as a guide to solve the target problem.

All three variants of source examples are one-step equations that have one operational line and two relational lines (Ngu and Phan, 2016, 2017). We place the three equations side-by-side to facilitate the mapping process (Kurtz et al., 2001; Rittle-Johnson and Star, 2009; Matlen et al., 2020). Furthermore, we label the solution steps—for example, Lines 1, 2, and 3 in Eq. 1 (Ngu and Phan, 2016, 2017) in order to provide explicit cue (Richland et al., 2017), which would encourage and facilitate active comparison. In essence, Eq. 1 is the main source example, whereas Eqs. 2 and 3 are derivatives of Eq. 1. The difficulty level of the three variants increases from Eqs 1–3. Eq. 1 differs from Eq. 2 in terms of the relative position of the pronumeral (i.e., left side vs. right side). Different orientation of the pronumeral would prevent direct alignment of the relational elements (Kurtz and Gentner, 2013; Matlen et al., 2020), and therefore this would adversely affect the efficiency of the analogical comparison. In contrast, displaying Eqs. 2 and 3 in the same orientation whereby the pronumeral is located in the right side of the equation would enable direct alignment of relational elements and, thus, this would facilitate the mapping process (Kurtz and Gentner, 2013). It should be noted that Eq. 2 (e.g., 2 = *x*/5) aligns with the target problem (e.g., sin30° = *x*/6), given that both problems have pronumerals that are located on the right side of the equation. Equation 3, in contrast, differs from Equation 2 because the former has a decimal number. The location of the pronumeral on the right side of the equation and the presence of a decimal number are considered as *special features* of one-step equations; these specific features, we contend, pose serious challenges for many students (Ngu and Phan, 2017).

In line with the recommendation by Kurtz et al. (2001) to facilitate analogical reasoning, our conceptualization requires that students complete three tasks (see Figure 1). Our aim is to encourage students to engage in deep processing of the three source examples. For the first task, students are required to compare and describe the similarities and differences between the three equations with respect to Line 1. The aim is to assist students to engage in deep analogical reasoning, leading to the identification of a common relational structure across the three equations. Comparing Eqs. 1 and 2, for example, would uncover the different location of the pronumeral (i.e., left side vs. right side). Comparing Eqs. 2 and 3, in contrast, would reveal that these equations do not exhibit a one-to-one correspondence in terms of the attribute of elements due to the presence of the decimal number in Eq. 3. It should be noted that Eq. 3 (1.2 = *x*/3) aligns with the target problem (sin30° = *x*/6), given that both problems have pronumerals located on the right side of the equation and that sin30° can be expressed as a decimal. Furthermore, as we can see, the mathematical operation for Line 1 (e.g., ÷ 2 becomes × 2 in Eq. 1) is the same across the three equations. Thus, having compared Line 1 of the three equations, we expect students to realize that these three equations belong to the same category of linear equations, thus requiring the use of the same mathematical operation to solve.

In relation to the second task, students are required to generate parallel solution steps, such as Lines 2 and 3 of Eqs. 2 and 3, which align with Lines 2 and 3 in Eq. 1 (Kurtz et al., 2001). Generating parallel solution steps for Eqs. 2 and 3 would draw students’ attention to a one-to-one correspondence with reference to the relational elements between the three equations. The third task, in contrast, requires students to answer a prompting question, for example: “Why might it be helpful to compare Eqs. 1–3?” We anticipate that such task would reinforce students’ understanding of the similarity between the three equations in terms of the schema for the shared solution procedure. In short, having completed the three tasks, we expect students to draw inference and recognize that the three equations shared a similar solution procedure, despite the relative position of the pronumeral (i.e., right side vs. left side) and the difference in the format of a number (e.g., 2 vs. 1.2). Once students have mentally represented the three variants of source examples and inferred a schema for the shared solution procedure, we expect them to select a relevant source example (1.2 = *x*/3) and subsequently to use this to solve the target Trigonometry problem (sin30° = *x*/6). This would constitute the second stage in the analogical learning process.

### Second Stage: Map a Relevant Source Example and the Target Problem

We envisage that students would have learned the definition of trigonometric ratios prior to their learning of how to solve Trigonometry problems that involve algebraic transformation skills. Each trigonometric ratio represents a number (i.e., a fraction or a decimal number), which is defined as one side over another side in a right-angled triangle.

**FIGURE 2:**
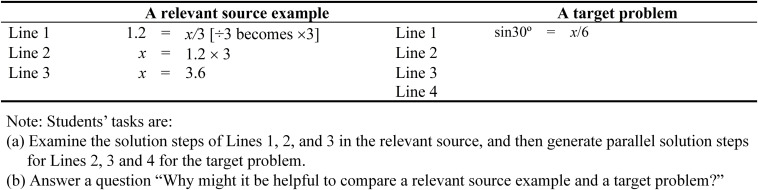
Solution procedure of a relevant source example and a target problem.

As shown in Figure 2, by placing a relevant source example (1.2 = *x*/3) and a target problem (sin30° = *x*/6) side-by-side, it is not necessary to provide a hint to access the relevant source example (Rittle-Johnson and Star, 2009; Matlen et al., 2020). Once again, we provide explicit cue (Richland et al., 2017) in which we use Lines 1, 2, 3 and so on to denote the solution procedure. For the first task, students are required to examine the solution steps of Lines 1, 2, and 3 in the relevant source example, and then generate parallel solution steps for the target problem, which are denoted by Lines 2, 3, and 4. On examining the target problem, we expect students to retrieve their prior knowledge of expressing sin30° in a decimal number and then complete Line 2 of the target problem. In doing so, the students are likely to notice a similarity between Line 1 of a relevant source example (1.2 = *x*/3) and the first solution step of the target problem (0.5 = *x*/6). Consequently, through mapping activities, we contend that this would guide the generation of solution steps for Lines 3 and 4 of the target problems, which are similar to the solution steps of Lines 2 and 3 of the relevant source example. Accordingly, from our point of view, the best alignment between the relevant source example and the first solution step of the target problem would occur as both share similar objects and relation (Richland et al., 2006).

Having generated the missing parallel solution steps for the target problem, students then proceed onto the second task. We recommend the use of open-ended questions as additional supporting cues for reflection, consolidation, and understanding—for example, “Why might it be helpful to compare a relevant source example and a target problem?” Reflection questions, we argue, may assist students to engage in deep processing of the relevant source example and the target problem (Rittle-Johnson and Star, 2007). Ultimately, having completed both tasks in Figure 2, we expect students to draw inference with reference to the schema for the shared solution procedure of a linear equation with a fraction (e.g., 1.2 = *x*/3) and the first solution step of Trigonometry problem (e.g., 0.5 = *x*/6), whereby sin30° in the target has been replaced with a decimal number.

### Summary

We propose a mental representation of three variants of source examples, leading to the selection of a relevant source example for the target problem. Our proposition differs from prior studies (Holyoak and Koh, 1987; Ngu and Yeung, 2012) that suggest a mental representation of one source example only. Existing recommendations highlight a one-to-one mapping between cases or examples to facilitate analogical learning (Alfieri et al., 2013; Goldwater and Schalk, 2016). Differing from existing recommendations, however, we emphasize a shared solution procedure between a relevant source example and the first solution step of the target problem (i.e., a subset of the target problem).

In line with the concept of learning by comparison (Rittle-Johnson et al., 2017), we place the relevant source example and the target problem side-by-side. We also label the solution procedure of the relevant source example as well as the missing parallel solution procedure for the target problem. Our aim here, in this analysis, is to draw students’ attention to the critical feature of the solution steps that constitutes a common structure between the relevant source and the target problem. Active analogical comparison would result when students generate the missing parallel solution steps for the target problems. The provision of a prompting question in conjunction with the generation of the missing parallel solution steps for the target problem would, from our point of view, assist students to deduce a schema of the shared solution procedure between the relevant source example and the first solution step of the target problem.

Overall, we contend that our proposed two major stages provide important insights, which may promote analogical learning: (i) a mental representation of three variants of source examples and then select a relevant source example among them, and (ii) perform mapping activities between a relevant source example and a target problem. We argue that our proposition, differing from existing research development, is informative for its structured sequencing, enabling students to construct their understanding into the solving of Trigonometry problems that involve algebraic transformation skills via the use of both learning by analogy and learning by comparison concepts.

Research on *expertise reversal effect* has placed emphasis on the specific *interaction* between an instructional method and a learner’s expertise in the relevant domain (Kalyuga et al., 2003). In brief, with a focus on the expertise reversal effect, it is noted that learners with varying levels of expertise would require different types of instructional methods. Accordingly, expert learners may not necessarily have to mentally represent three variants of source examples and select a relevant source example, and/or mentally represent the target problem plus its first solution step. With in-depth knowledge and understanding of linear equations and trigonometric ratios, expert learners may realize that sin20° = *x*/6 is similar to 3 = *x*/8. Once they realize that sin20° is a decimal number, they would have a solution for sin20° = *x*/6. Indeed, noticing a similarity between sin20° = *x*/6 and 3 = *x*/8 would result in expert learners retrieving a learned solution procedure for the solving of 3 = *x*/8, which could then be used to solve sin20° = *x*/6.

The theoretical rationale that explains the procedure of solving Trigonometry problems with pronumerals as a numerator could be applied to Trigonometry problems that have pronumerals as a denominator, given that both types of Trigonometry problems are related to linear equations with a fraction. In the next section, we explore in detail the solving of Trigonometry problems that have pronumerals as a denominator.

## Trigonometry Problems With a Pronumeral as the Denominator

**FIGURE 3:**
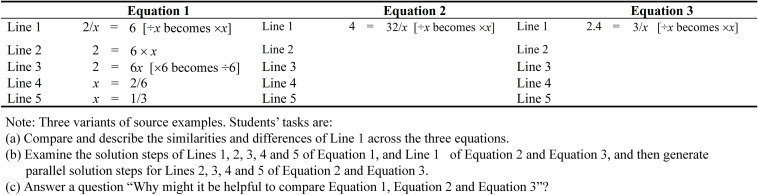
Three variants of source examples.

**FIGURE 4:**
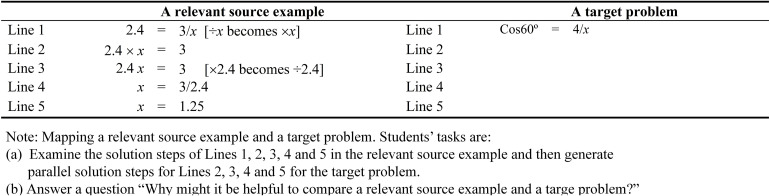
Mapping a relevant source example and a target problem.

As noted earlier, the relative location of the pronumeral (i.e., numerator vs. denominator) determines the complexity of the Trigonometry problem. Differential solution steps favor Trigonometry problems that have pronumerals as a numerator. More specifically, Trigonometry problems that have pronumerals as a denominator are more complex than Trigonometry problems that have pronumerals as a numerator. In this analysis, the former has more operational lines (2 vs. 1) and relational lines (3 vs. 2) when compared to the latter (see Figures 2, 4). The rationale for promoting analogical learning for the two types of Trigonometry problems that differ in the location of pronumeral (i.e., numerator vs. denominator), likewise, is the same. Therefore, similar to the case of learning how to solve trigonometry problems with pronumerals as a numerator (e.g., sin30° = *x*/6), we argue that learning to solve cos60° = 4/*x* would require learners to engage in the following: (i) mentally represent three variants of source examples, and then select a relevant source example from these source examples (Figure 3), (ii) mapping a relevant source example and a target problem (Figure 4).

The three variants of source examples are one-step linear equations that have two operational lines and three relational lines (Figure 3). Equations 1, 2 are similar except the location of the pronumeral (left side or right side). For Eq. 2, the location of the pronumeral is on the right side of the equation (4 = 32/*x*), which is similar to the location of the pronumeral for the target problem (Cos60° = 4/*x*) (Kurtz and Gentner, 2013). Equations 2 and 3 are similar with the exception of a decimal number for the latter. As noted earlier, the presence of special features (e.g., a pronumeral located on the right side of the equation, a decimal number, etc.) contributes to the complexity of one-step equations. Accordingly, the three variants of the linear equations increase in complexity from Eqs. 1–3. It should be noted that the rationale of completing the tasks in Figures 3, 4 for learning Trigonometry problems with a pronumeral as a denominator is similar to the rationale of completing the tasks in Figures 1, 2 for learning Trigonometry problems with a pronumeral as a numerator. Thus, we will not discuss the tasks in Figures 3, 4 separately here.

An inspection of the solution procedure for the two Trigonometry problem types (i.e., pronumeral as a numerator vs. pronumeral as a denominator) suggests that there are a few notable differences. As noted earlier, for example, differential number of relational (3 vs. 4) and operational (1 vs. 2) favors Trigonometry problems that have pronumerals as a numerator (Ngu and Phan, 2016). Hence from this disparity, we argue that learning to solve Cos60° = 4/*x* would pose a greater challenge than learning how to solve sin30° = *x*/6 (i.e., see Figure 2 vs. Figure 4). However, having said this, we contend that prior knowledge (e.g., algebraic transformation knowledge) would help a learner to reduce the number of relational lines. For example, referring to Figure 4, a learner may skip Line 2 of the relevant source example (i.e., 2.4 × *x* = 3) and the corresponding Line 3 of the target problem (i.e., 0.5 × *x* = 4). It should be noted that expert learners may also recognize and realize that cos40° = 5/*x* and 3 = 12/*x* are similar to each other. Once they realize that cos60° is a decimal number (i.e., 0.5), they would have recognized that the same method could be used to solve both problems.

How can we help learners to distinguish the two types of trigonometry problems: a pronumeral as a numerator (e.g., sin30° = *x*/6) vs. a pronumeral as a denominator (e.g., cos60° = 4/*x*)? Previous research has indicated that secondary school students performed better when the pronumeral is a numerator rather than that of a denominator (Kendal and Stacey, 1998; Weber, 2005). The number of operational and relational lines, as we have argued, reflects the complexity of the solution procedure. As noted previously, the Trigonometry problems that have pronumerals as a numerator have fewer operational (e.g., 1 vs. 2) and relational (3 vs. 4) lines than Trigonometry problems that have pronumerals as a denominator.

## Differentiate the Two Types of Trigonometry Problems

**FIGURE 5:**
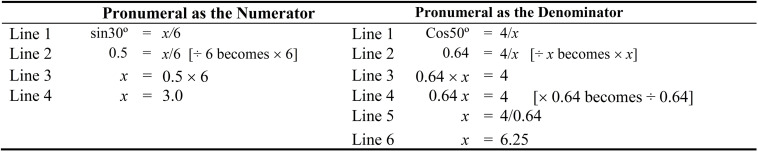
A comparison between solution procedure of two types of trigonometry problems.

The concept of learning by comparison, from our point of view, may assist learners to distinguish the two types of Trigonometry problems. We propose to place the two types of Trigonometry problems side-by-side and instruct learners to identify the similarities and differences between them (Rittle-Johnson et al., 2017). For example, with reference to Figure 5, we could ask learners to indicate major similarities and/or differences. From our inspection, there are a number of possibilities: (i) the location of the pronumeral (i.e., a numerator vs. a denominator), (ii) sin30° is similar to cos50°, both of which are decimal numbers, (iii) once we replace sin30° or cos50° with a decimal number, it becomes a linear equation with a fraction (e.g., Figures 2, 4), (iv) differential number of relational lines (i.e., 2 vs. 3) and operational lines (i.e., 1 vs. 2) favors a Trigonometry problem that has a pronumeral as a numerator, and (v) the inverse method is used to solve both types of Trigonometry problems. Once learners have compared and identified the similarities and differences between the two types of Trigonometry problems, we predict that they would have noticed differential algebraic transformation skills involved in solving these two types of Trigonometry problems.

**FIGURE 6:**
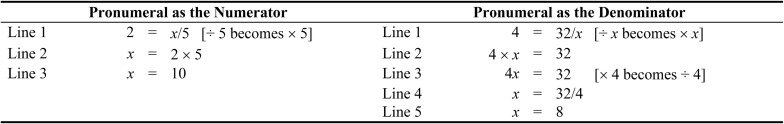
A comparison between an equation with pronumeral as numerator and an equation with pronumeral as denominator.

For novice learners, in contrast, we argue that a basic step for understanding, it would be ideal to compare linear equations with a fraction side-by-side in order to identify their similarities and/or differences (see Figure 6). One notable characteristic for identification, in this case, relates to the location of the pronumeral (i.e., as a numerator vs. a denominator), which influences the algebraic transformation skills involved in solving these two types of linear equations. Learning and mastering this basic step, we contend, may facilitate understanding of Trigonometry problems that have pronumerals as both numerator and denominator. For example, a comparison of cos60° = 2/*x* and cos60° = *x*/2 side-by-side indicates that one main difference lies in the location of the pronumeral—that is, 2/*x* vs. *x*/2. This identification would, in turn, prepare novice learners to solve both types of Trigonometry problems—in this case, sin30° = 8/*x* vs. cos60° = *x*/2.

## Discussion

Trigonometry, indeed, is a difficult topic for many secondary students, especially when we confound Trigonometry problems with the location of the pronumeral (i.e., a numerator vs. a denominator) (Kendal and Stacey, 1998). We argue that it is possible to counter this pervasive issue by considering the use of learning theories—in this case, learning by analogy and learning by comparison concepts (Kurtz et al., 2001; Rittle-Johnson and Star, 2007; Alfieri et al., 2013). Our conceptualization, as detailed in the preceding sections, proposed a mental representation of three variants of source examples. Of these three variants of source examples, we select one relevant source example for the target problem. We highlight the mapping of a relevant source example and the first solution step of the target problem in order to achieve optimal alignment between these two problems. Our proposition, in its totality, has advanced the study of learning by analogy for its deliberation on a relevant source example from three variants of source examples. This pedagogical contention is different from previous research (e.g., Holyoak and Koh, 1987), which places emphasis on the use of one source example. Moreover, we emphasize a subset of the target problem and not the whole target problem for the purpose of implementing a one-to-one mapping task between the relevant source example and the first solution step of the target problem. Therefore, we recommend a comparison between a source example and a subset of a target problem to facilitate analogical learning.

At the same time, capitalizing on the significance of learning by comparison, we consider the use of comparison within the context of Trigonometry problems for their similarities and differences. Our conceptualization, which to date researchers have not studied, is innovative for its emphasis on the simultaneous comparison of different types of Trigonometry problems. This comparison of two types of Trigonometry problems side-by-side, in particular, seeks to overcome the long-standing difficulty of learning Trigonometry problems that differ because of the relative position of the pronumeral (i.e., numerator vs. denominator). With this in mind, we urge educators to consider the use of the instructional practices that help students to recognize and understand the two main Trigonometry problem types.

**FIGURE 7:**
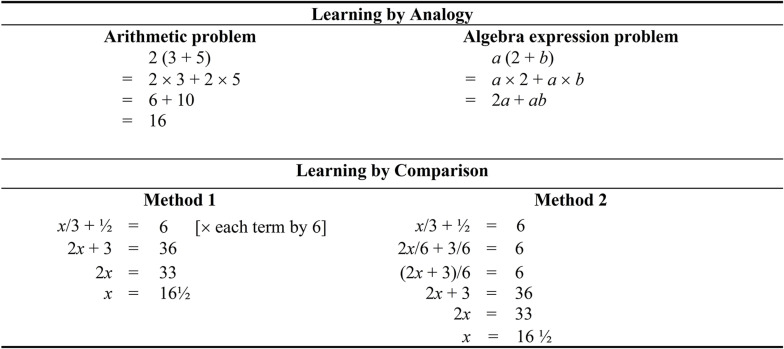
Examples of mathematics learning via learning by analogy and learning by comparison.

How can the concepts of learning by analogy and learning by comparison assist us in our pedagogical practices in other areas of mathematics? Consider in this case the learning of *Algebra expression problems*, which is presented in Figure 7. The focus of understanding, in this case, is related to our previous mentioning of comparison—that is, a parallel comparison is made between “2(3 + 5)” and “*a*(2 + *b*).” Our postulation is that *alignment of relational elements* may assist learners to understand the logic of manipulating variables. For example, as shown, 2*a* simply means that 2 is multiplied by *a* (a variable). From this consideration, in a secondary school, a student may compare the two equations side-by-side and deduce that 2 is equaled to *a*, and 5 is equaled to *b*. In a similar vein, we contend that it is of value to consider learning by comparison as an instructional tool, which could facilitate the learning of *linear equations*. As a point of comparison of linear equations that have a fraction (e.g., Figure 7), for example, we note that having fewer solution steps (Method 1) is more beneficial as this would impose lower cognitive load (Ngu et al., 2018).

In conclusion, as educators, we recognize the important topic of Trigonometry. Moreover, from our professional experiences, we acknowledge that there is a pervasive issue when Trigonometry problems have pronumerals that operate as both a numerator and a denominator. This distinction (i.e., pronumeral as a numerator vs. pronumeral as a denominator), we contend, is relatively unique, confounding the difficulty of students’ understanding of how to solve different types of Trigonometry problems that involve algebraic transformation skills. From our existing empirical research and other researchers’ inquiries and findings, we derived a pedagogical conceptualization that could assist students to understand the complexity of Trigonometry problems. In this analysis, considering the effectiveness of both learning by analogy and learning by comparison, we proposed an alternative sequence of steps for students to follow. We recommend educators to implement and explore the potentiality of our proposition when teaching two types of Trigonometry problems that differ in terms of the relative location of the pronumeral (i.e., numerator vs. denominator).

## Author Contributions

BN and HP were responsible for the conceptualization and write-up of this manuscript. Both authors contributed to the article and approved the submitted version.

## Conflict of Interest

The authors declare that the research was conducted in the absence of any commercial or financial relationships that could be construed as a potential conflict of interest.
